# Risk factors and prediction model for low-birth-weight infants born to women with gestational diabetes mellitus

**DOI:** 10.3389/fpubh.2024.1432033

**Published:** 2024-10-10

**Authors:** Yu-qing Pan, Xin-xin Huang, Xiu-min Jiang

**Affiliations:** ^1^School of Nursing, Fujian Medical University, Fujian Province, China; ^2^Fujian Maternity and Child Health Hospital, College of Clinical Medicine for Obstetrics & Gynecology and Pediatrics, Fujian Medical University, Fuzhou, Fujian, China

**Keywords:** gestational diabetes mellitus, low birth weight infant, nomogram, prediction model, risk factor

## Abstract

**Aim:**

To explore the risk factors for low-birth-weight infants born to pregnant women with GDM to develop a prediction model and to construct a prediction nomogram for the risk of low birth weight infants born to pregnant women with GDM.

**Methods:**

The clinical data of singleton infants diagnosed with gestational diabetes mellitus in Southeast China in 2019 were retrospectively reviewed. Gestational conditions and neonatal weight were assessed.

**Results:**

A total of 386 diabetic mothers and infants were enrolled, including 193 in the low birth weight group and 193 in the normal birth weight group. The statistically significant factors were age over 36 years (OR = 1.916, 95% CI 1.048–3.505), junior high school education (OR = 4.454, 95% CI 1.882–10.543), history of fetal distress (OR = 0.120, 95% CI 0.016–0.925), gestational hypertension (OR = 3.681, 95% CI 1.357–9.986), preeclampsia (OR = 24.652, 95% CI 5.956–102.036), threatened preterm birth (OR = 18.393, 95% CI 8.457–39.999), triglycerides (OR = 0.642, 95% CI 0.485–0.850), and inadequate gestational weight gain (OR = 1.997, 95% CI 1.162–3.432). The area under the receiver operating characteristic curve (AUC) was 0.834 (95% CI: 0.794–0.874, *p* < 0.001), and the sensitivity and specificity were 82.38 and 87.56%, respectively. The goodness-of-fit test likelihood ratio 2 was 2.089 (*p* = 0.978). The comprehensive nomogram model showed that the discrimination and mean absolute error were 0.834 and 0.015, respectively. The calibration curves showed acceptable agreement between the predictions of the column line plots and the observations. The DCA curves showed good positive net yields in the prediction model.

**Discussion:**

This study established a prediction model and risk score for low birth weight in pregnant women with GDM. It helps pregnancy clinics to identify the risk of low birth weight in newborns promptly, in addition to glycemic control and weight management for pregnant women with GDM, and should improve the appropriate treatment plan for pregnant women with higher risk, to provide personalized and precise treatment for pregnant women with GDM and improve infant outcomes.

## Introduction

Gestational diabetes mellitus (GDM) is the first diagnosis of any degree of abnormal glucose metabolism during pregnancy (1) and is usually detected during the second trimester (24–28 weeks) or the third trimester (28 weeks and beyond). GDM is a major public health problem for women worldwide (2). However, the adverse consequences of GDM can be actively prevented and reversed. Through clinical experience, early diagnosis, and treatment of GDM, opportunities arise for clinical intervention and the mitigation of adverse perinatal outcomes (3).

Fetuses receive increased amounts of glucose from mothers with GDM during pregnancy, which promotes insulin secretion and increases fetal growth. Newborn birthweight (NBW) refers to newborn birthweight within 1 h after delivery (4) and is one of the most direct indicators of newborn health. Low birth weight included small for gestational age (SGA) and low birth weight (LBW). Low birth weight increases perinatal morbidity and mortality, including neonatal asphyxia, hypothermia, and abnormal nervous system development, and even increases the risk of future preeclampsia, pregnancy-induced hypertension, and SGA, as well as significantly increases the incidence of mental diseases in women with LBW (5). At the same time, the treatment of poor birth weight and its complications not only causes a large economic burden in the long and short term for the country and society but also increases additional nursing costs (6). Newborn birth weight has become the focus of global public health. Early identification of abnormal fetal weight and timely nursing and health education are highly important for ensuring maternal and offspring health.

Epidemiological studies have shown that infants born to mothers with GDM have a greater rate of adverse perinatal outcomes (e.g., asphyxia, hypoglycemia, and birth defects) than infants born to mothers without GDM (7). Current studies on the birth weight of infants born to diabetic mothers mostly focus on macrosomia. Researchers such as Naha (8), Tomlinson (9), and others have constructed prediction models for macrosomia and large for gestational-age infants born to pregnant women with GDM. Previous studies have shown that pregnant women with GDM are more likely to have large gestational age infants and macrosomia than pregnant women without GDM (10), and some studies have shown that mild GDM is also considered to be related to a reduction in neonatal birth weight after treatment (11). A cross-sectional study conducted in 2021 at Jamhouria Hospital/Neonatal Ward found that gestational diabetic and pre-pregnant diabetic mothers delivered 16.7% of newborns with LBW and 13.3% with macrosomia (12). Prior research has demonstrated that there is no statistically significant variance in the occurrence of LBW and small for SGA between women afflicted with GDM and those who are not (13). The incidence rates display a tendency to rise with time (14). Consequently, GDM does not mitigate the probability of pregnant women delivering infants with LBW. Clinically, a uniform approach to nutritional management, blood sugar control, and health education for pregnant women with gestational diabetes is aimed at reducing the occurrence of adverse pregnancy outcomes. Quite the contrary, when expectant mothers with GDM undergo rigorous dietary, physical activity, or insulin interventions, it may exacerbate the severity of LBW (8). Therefore, building a risk prediction model can help clinicians identify high-risk GDM pregnant women for delivering LBW babies in the early or mid-pregnancy. This helps to take early intervention measures, such as adjusting dietary structure, to improve pregnancy outcomes and reduce the incidence of LBW babies. However, thus far, few scholars have developed a risk prediction model solely targeting the LBW of GDM newborns as an outcome indicator.

The nomogram model serves as a computational graphical device that substitutes intricate mathematical formulations, effectively portraying the outcomes of regression analyses in an intuitive and visually comprehensible manner. This approach has garnered widespread adoption across diverse medical disciplines. The nomogram inherently accommodates the nuanced impact of each influencing factor on the clinical outcome, assigning distinct scores to each, where the cumulative score serves as a proxy for the risk of a particular clinical event. In clinical practice, physicians can expeditiously evaluate a patient’s risk level by leveraging the information encoded within the nomogram, thereby facilitating informed clinical decision-making. Consequently, the development of a nomogram-based risk prediction model in this study presents itself as a streamlined tool, empowering healthcare providers to swiftly assess the risk of delivering LBW infants among GDM pregnant women and tailor personalized treatment strategies accordingly.

Therefore, accurate identification of pregnant women with GDM who may have poor birth weight and individualized intervention and management is very important to improve the occurrence of maternal and child complications and ensure the safety of the mother and child. To date, there is no relevant study on LBW infants born to mothers with GDM in China. In this study, we sought to investigate the demographic and clinical characteristics and risk factors for LBW infants born to mothers with GDM in China to provide a basis for timely identification of risk factors for neonatal underweight during pregnancy, implementation of intervention treatment for pregnant women with GDM, and improvement of neonatal prognosis.

### Research design

This was a retrospective case–control study.

### Research objects and methods

#### Research site

A tertiary hospital in southeast China.

### Research subjects

Pregnant women with GDM and their newborns who gave birth during regular antenatal check-ups at the research hospital from January 1, 2019, to December 31, 2019, were included. Relevant data during pregnancy and the neonatal period were collected. LBW was included in the case group, and normal birth weight was included in the control group. The sample size of the control group and the case group was 1:1. The gestational age of the control group was matched by the tendency scoring method, and the matching tolerance was set at 0.1.

### Diagnostic criteria

The diagnostic criteria for GDM were as follows: according to the 2014 Chinese Guidelines for the Diagnosis and Treatment of Gestational Diabetes Mellitus, a 75 g OGTT (one-step method) was performed at 24–28 weeks of gestation, and the blood glucose value met any one or more of the following criteria: fasting blood glucose at least 5.1 mmol/L; OGTT 1 h blood glucose at least 10.0 mmol/L; and OGTT 2 h blood glucose at least 8.5 mmol/L (15)^.^

### Weight measurement

The weight and height of the participants were measured using standard procedures in units of 0.1 kg on a digital scale and 0.1 cm on a stadiometer, respectively, while participants wore light clothes and were barefoot. Prepregnancy weight was self-reported at the first prenatal visit and measured at the second and third-trimester prenatal visits.

### Inclusion and exclusion criteria

The inclusion criteria were as follows: ① aged ≥18 years; ② pregnant woman diagnosed with gestational diabetes at 24–28 weeks; ③ pregnant woman who underwent prenatal examination and gave birth at the study hospital; and ④ single live birth.

The exclusion criteria were as follows: ① prepregnancy diabetes mellitus; ② incomplete electronic medical record information; ③ newborn suffering from congenital defects and other perinatal diseases that seriously affect growth and development; ④ polycystic ovarian syndrome before pregnancy; ⑤ pregnant women who had received medical treatment for chronic conditions, such as oral glucocorticoids, thiazide diuretics, beta-blockers, ACE inhibitors, or antiretroviral drugs; and ⑥ late abortion or induced labor due to fetal abnormalities.

### Sample size

The sample size was calculated using PASS 15.0. According to the preliminary survey, the proportion of patients with inadequate GWG in the LBW group was 55%, and the percentage of patients with exposure to risk factors in the normal weight group was 37%. The level of significance was set at 5%, and the statistical power was set at 1–*β* =0.90. The sample size of each group was 156, and the minimum sample size was 312. The number of samples was increased by 10% to account for incomplete data, and a minimum sample size of 347 participants was needed.

### Variable definition

Prepregnancy BMI was defined as prepregnancy weight (kilograms) divided by prepregnancy height squared (meters). Prepregnancy BMI status was categorized into 4 levels for the Chinese population as follows: underweight, < 18.5 kg/m^2^; normal weight, 18.5–22.9 kg/m^2^; overweight, 23–24.9 kg/m^2^; and obese, ≥25 kg/m^2^ (16).

According to American Institute of Medicine standards, prepregnancy underweight (BMI <18.5 kg/m^2^) pregnancy weight gain is appropriate for 12.5 ~ 18.0 kg; prepregnancy weight normal (18.5 kg/m^2^ ≤ BMI < 25.0 kg/m^2^) pregnancy weight gain is suitable for 11.5 ~ 16.0 kg; prepregnancy overweight (25.0 kg/m^2^ ≤ BMI < 30.0 kg/m^2^) pregnancy weight gain is 7.0 ~ 11.5 kg; and prepregnancy obesity (BMI ≥ 30.0 kg/m^2^) is suitable for pregnancy weight gain of 5.0 ~ 9.0 kg (17). If the weight gain during pregnancy is less than or greater than the appropriate amount, there is insufficient weight gain during pregnancy and excessive weight gain during pregnancy, respectively.

LBW was defined as <2,500 g at birth. Preterm birth was defined as a gestational age < 37 weeks at birth.

### Research tools/observation indicators

General and basic information, drug use, and physiological indicators were obtained from the hospital’s electronic medical records system.

The indicators included in the study included: age, ethnic groups, place of residence, degree of education, occupation, marital status, medical insurance, parity, order of birth, family history, history of miscarriage, history of macrosomia, history of GDM, history of fetal distress, history of premature rupture of membranes. Artificially assisted reproduction, intrahepatic cholestasis of pregnancy, hypertensive disorders of pregnancy, and hyperthyroidism. Hypothyroidism, anemia, threatened preterm labor, polyhydramnios, oligohydramnios, insulin, prepregnancy BMI, pregnancy weight gain, urinary ketone in early pregnancy, urinary ketone in the second trimester, OGTT-0h, OGTT-1h, OGTT-2h, triglycerides in early pregnancy, fasting blood glucose in the first trimester, 2 h postprandial blood glucose in early pregnancy, triglycerides in the second trimester, fasting blood glucose in the second trimester, 2 h postprandial blood glucose in the second trimester, glycated hemoglobin in the second trimester, average weekly abdominal circumference growth rate, the average weekly growth rate of uterine height.

### Data analysis

IBM SPSS 27.0 software was used for data analysis. Continuous variables were defined using the mean and standard deviation (SD) after assessing the symmetry of distributions through the observation of histograms. In instances where medians and percentiles P_25_ and P_75_ were presented, this was due to the absence of symmetry. To evaluate the normal distribution of continuous variables, we examined histograms, symmetry, and kurtosis, taking into account the size of our sample. Categorical variables were defined by their total number and frequency (%). Age was modified as a dichotomous variable using advanced pregnancy as the cut-off point. For the variable inferential analysis, normally distributed measurement data were analyzed by two independent sample *t*-tests or analysis of variance for comparisons between groups. Continuous non-normally distributed variables and categorical variables were analyzed by nonparametric Kruskal–Wallis, and Mann–Whitney *U* tests. For categorical variables, we used the χ^2^ test or Fisher’s exact test (dichotomic variables and ≥20% cells with expected count <5). To assess associations, we used binomial and multinomial logistic regression to obtain crude and adjusted odds ratios (ORs) with 95% confidence intervals (CIs). The nomogram was constructed and drawn using R software version 4.1.2. The C-statistics between the nomograms and each of the independent predictor variables were compared using the Delong test. Calibration curves were used to analyze the agreement between the predictions of the column line plots and the actual observations. Decision curve analyses (DCA) were performed to assess the clinical utility of the predicted column-line plots by estimating the net benefit under the threshold probabilities of LBW types.

All tests with statistical significance were bilateral, and a *p*-value <0.05 was considered to indicate statistical significance.

## Results

**Figure 1:**
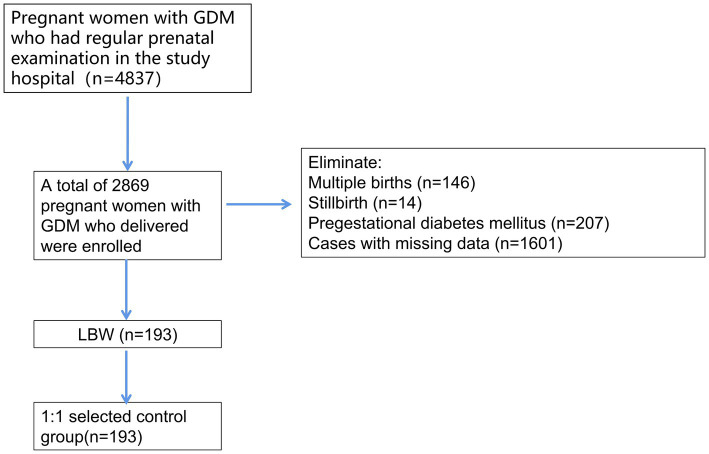
Technical road map.

A total of 4,837 pregnant women with GDM were identified in the electronic database, and 2,869 pregnant women with GDM met the inclusion and exclusion criteria after excluding 146 women with multiple births, 14 cases of stillbirth, 207 cases of pregestational diabetes, and 1,601 cases with missing data. A total of 193 cases of neonatal LBW were found, and the incidence of LBW was 6.73%. A total of 193 normal-weight infants born to pregnant women with normal GDM in the same period after PSM1:1 were selected as the control group and the corresponding technical roadmap is shown in Figure 1.

Univariate analysis revealed that age, education level, history of preterm birth, history of fetal distress, hypertensive disorders complicating pregnancy, threatened preterm birth, average weekly abdominal circumference growth rate and weight gain during pregnancy were significantly different (*p* < 0.05), as shown in Table 1.

**Table 1:** Factors associated with LBW for pregnant women with GDM [N(%)/X ± SD/M(P_25_, P_75_)].

	LBW (*n* = 193)	Normal weight (*n* = 193)	***χ****^2^*/t/*Z*	*P*
Age				
18 ~ 35	140(72.5)	159(82.4)	5.357	0.021
≥36	53(27.5)	34(17.6)		
Degree of education				
Junior high school and below	35(18.1)	10(5.2)		<0.001
High school	61(31.6)	79(40.9)	16.447	
University and above	97(50.3)	104(52.9)		
History of fetal distress				
No	191(99.0)	184(95.3)	4.585	0.032
Yes	2(1.0)	9(4.7)		
Hypertensive disorders of pregnancy				
No	152(78.8)	181(93.8)	18.393	<0.001
Hypertension in Pregnancy	41(21.2)	12(6.2)		
Threatened preterm labor				
No	115(59.6)	182(94.3)	65.553	<0.001
Yes	78(40.4)	11(5.7)		
Pregnancy weight gain				
Underweight gain	127 (65.80)	88 (45.60)	-----	<0.001
Normal weight gain	16(8.29)	22(11.40)		
Hyper weight gain	50(25.91)	83(43.01)		
Average weekly abdominal circumference growth rate	0.69(0.52–0.91)	0.75(0.63–0.93)	−2.412	0.016

Considering the importance of triglycerides during pregnancy on birth weight (12), triglycerides were included in the regression analysis as a potential independent variable. Collinearity diagnosis was performed on latent variables and variables with significant univariate analysis results (*p* < 0.05), and the results showed that all variables did not have collinearity and could be included in multivariate logistic regression analysis. With neonatal LBW as the dependent variable (1 = LBW, 0 = normal birth weight), backward regression was used to screen variables, and *p* = 0.15 was the exclusion criterion. The results showed that age ≥ 36 years, middle school and below education, threatened preterm labor, gestational hypertension, and excessive weight gain during pregnancy were risk factors for LBW born to GDM women. Compared with that of women 18–35 years old, the risk of LBW delivery was 1.795 times greater in GDM women ≥36 years old. Compared with women with a college education or above, the risk of LBW delivery was 4.424 times greater for GDM women with a junior high school education or below. Compared with those without preterm birth, the risk of LBW delivery was 18.073 times greater in those with preterm birth. The risk of LBW delivery was 7.829 times greater in those with pregnancy-induced hypertension than in those without pregnancy-induced hypertension. Compared with those who gained a normal weight during pregnancy, the risk of LBW delivery was 2.031 times greater in those who gained less weight during pregnancy. Triglycerides during pregnancy and a history of fetal distress were protective factors. For every 1 mmoL/L increase in triglycerides in early pregnancy, the risk of LBW delivery in GDM women decreased 1.538 times. The risk of LBW delivery was 8.065 times greater for GDM women with no previous history of fetal distress than for those with a history of fetal distress, as shown in Table 2.

**Table 2:** Multiple regression analysis for the factors associated with LBW among pregnant women with GDM.

Determinant	β	S.E	Wals χ2	P	OR	95%CI	
≥36 years old	0.585	0.305	3.677	0.055	1.795	0.987	3.263
Junior high school and below^a^	1.487	0.438	11.525	0.001	4.424	1.875	10.439
High school^a^	−0.174	0.269	0.416	0.519	0.841	0.496	1.425
A history of fetal distress	−2.084	1.035	4.055	0.044	0.124	0.016	0.946
Gestational hypertension^b^	2.058	0.423	23.661	<0.001	7.829	3.417	17.940
Threatened preterm labor	2.894	0.392	54.428	<0.001	18.073	8.377	38.992
Triglycerides in early pregnancy	−0.430	0.141	9.263	0.002	0.650	0.493	0.858
Insufficient weight gain during pregnancy^c^	0.709	0.275	6.625	0.010	2.031	1.184	3.484

^aUniversity education or above as reference; bNo hypertensive diseases during pregnancy as reference; cappropriate weight gain during pregnancy as reference.^

### Evaluation of discrimination

**Figure 2:**
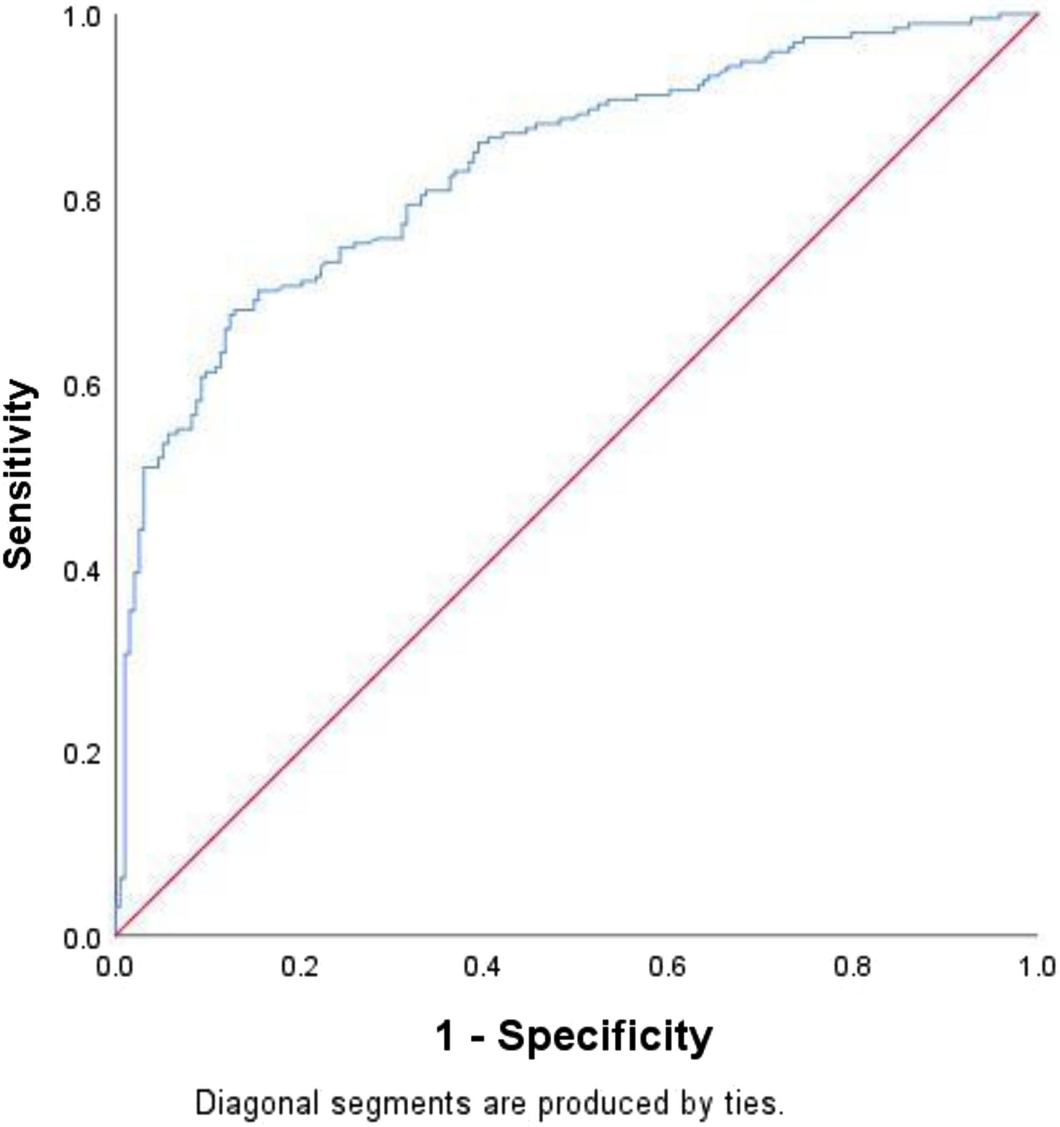
ROC curve plots. The sensitivity is the abscissa, and the (1-specificity) is the ordinate.

The ROC curve (Figure 2) was constructed by using the predicted probability of logistic regression as the state variable and LBW as the test variable, and the AUC value was 0.829 (95% CI: 0.789–0.870, *p* < 0.001), indicating that the diagnostic value was good. When the optimal cutoff value of the model was 0.464, the sensitivity and specificity were 70.46 and 82.90%, respectively.

#### Evaluation of calibration

The Hosmer–Lemeshow goodness of fit test was used to evaluate the calibration ability of the prediction model. The likelihood ratio 2 was 0.353, and the *p* value was 0.553, indicating that there was no significant difference between the predicted value and the observed value, and the calibration degree of the model had a good degree of fit.

#### Nomogram

**Figure 3:**
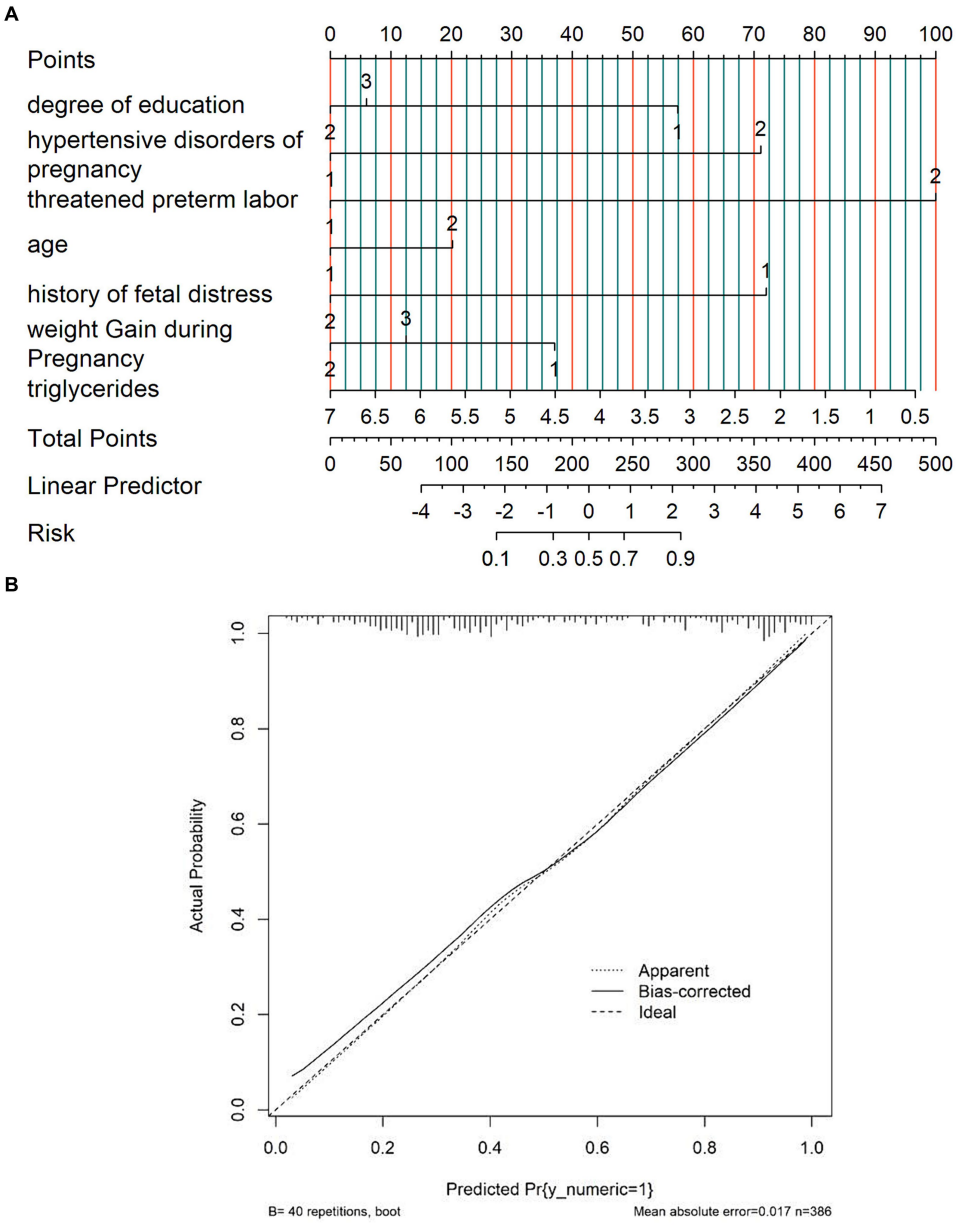
Nomogram and calibration curve for the incidence risk of low-birth-weight infants born to women with gestational diabetes mellitus in China: **(A)** nomogram and **(B)** calibration plot.

Based on the results of the multivariable analyses, a nomogram for the risk of LBW resulting from GDM was constructed, as shown in Figure 3. Each value of these variables yields a score on the score axis. Each score can easily be added up to create a total score, and by extrapolating the total score to the entire score scale, the likelihood of LBW can be calculated. Pregnancy-induced hypertension was found to have the greatest impact on the prediction of LBW.

**Figure 4:**
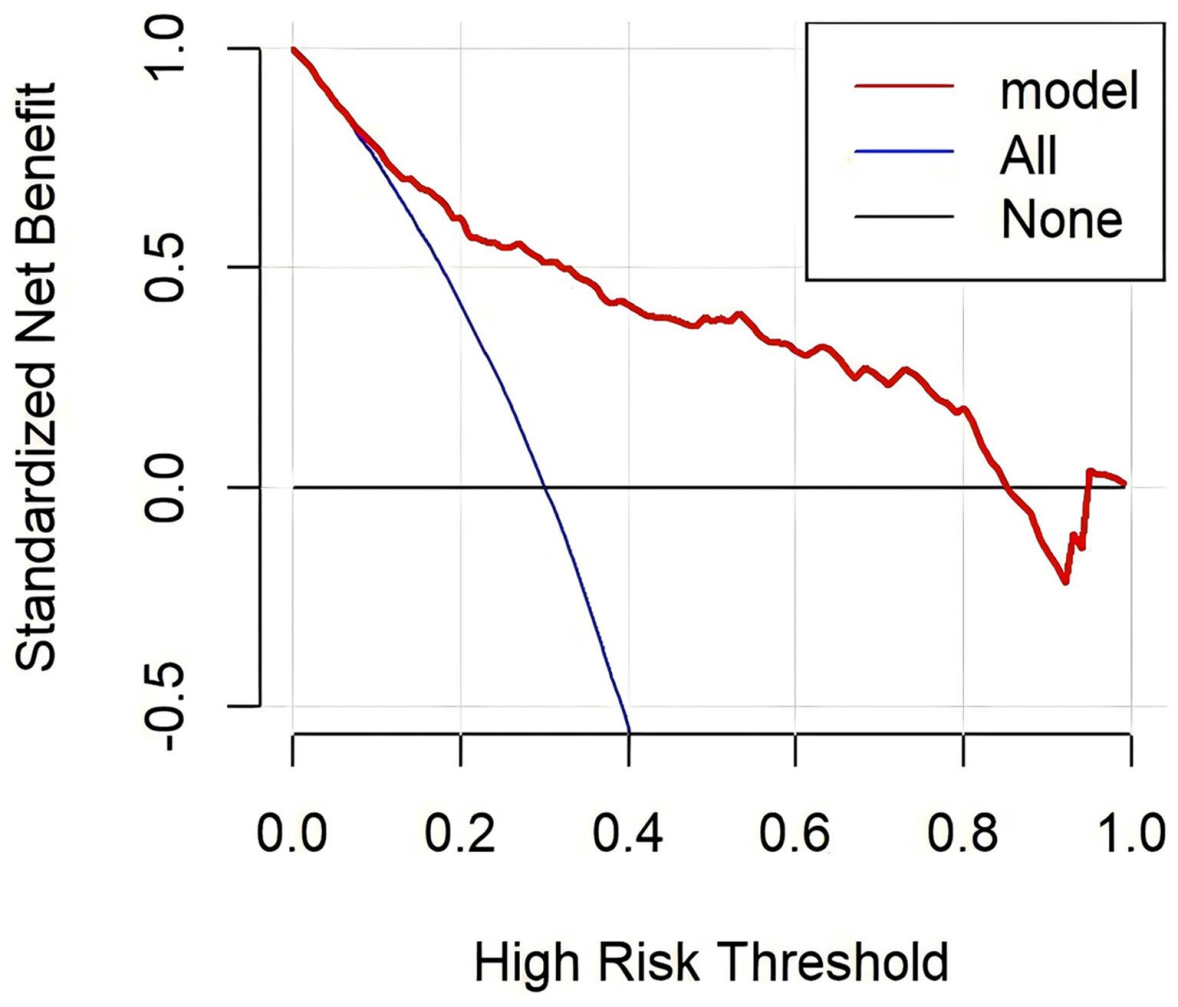
Decision curve analysis plot of the nomogram.

The calibration curves overlap the ideal line, indicating that the actual probabilities are in good agreement with the LBW probabilities predicted by the column plot. The discrimination of the nomogram yielded a c-index of 0.834. The mean absolute error of the calibration curve of the nomogram was 0.015 (Figure 3). The DCA plot showed good positive net benefits in the predictive nomogram model for majority threshold probabilities (Figure 4).

## Discussion

### Principal findings

This is a study of a prediction model and risk score for LBW infants who were born to pregnant women with GDM in Southeast China. The risk score includes several factors during pregnancy.

With the exposure to long- and short-term harm caused by the poor birth weight of neonates born to pregnant women with GDM, related research on the influencing factors of LBW has become a focus. The results of this study showed that the LBW of pregnant women with GDM was mainly related to basic conditions during pregnancy, adverse pregnancy history, insufficient weight gain during pregnancy, and hypertensive disorders during pregnancy.

### Clinical implications

#### Demographic data

We found that the risk of LBW was greater in pregnant women with GDM who had a junior high school education or less than in those with a college education or above. The educational level can be used as an indicator of individual socioeconomic status to a certain extent and is usually an important determinant of diet, living status, and health care during pregnancy (18). Studies have shown that those with lower education levels have fewer prenatal visits (19), poor awareness of health care during pregnancy, and a greater likelihood of delivering LBW.

Age is an important influencing factor of LBW in pregnant women with GDM. Under the influence of various social factors, such as the mature application of reproductive technology, the gradual increase in women’s education level, and occupational demand, the number of women of advanced maternal age is gradually increasing, but this population faces greater pregnancy risk. Therefore, the role of health education cannot be ignored. It is necessary to disseminate knowledge about the increased incidence of adverse maternal and infant outcomes in older adult women. At the same time, it is necessary to further improve the prenatal examination system so that all pregnant women can receive standardized and systematic prenatal screening in early pregnancy to ensure the health of mothers and children.

### Hypertensive disorders of pregnancy

The presence of hypertensive disorders complicating pregnancy is an important factor for predicting LBW in women with GDM. At present, a large number of international conclusions support that gestational hypertension and preeclampsia are risk factors for LBW (20), which is consistent with our results. The reason should be that GDM in pregnant women complicated with hypertensive disorders during pregnancy may affect the activity of glycogen glycolysis enzymes in the placenta but make it difficult for the fetus to obtain oxygen and nutrition, thus affecting the growth and development of the fetus (21) and eventually causing pregnant women with GDM to experience LBW. Especially for pregnant women with GDM and preeclampsia, glucose control is more difficult, resulting in poor glucose control. At the same time, placental small vessel spasm significantly reduces placental blood perfusion, causes placental function decline, and eventually increases the incidence of LBW. Therefore, in addition to close monitoring of blood pressure during pregnancy, health education during pregnancy should be increased. Yue et al. showed that there is an interaction effect between health education during pregnancy and gestational hypertension. Health education for pregnant women with hypertension can reduce the incidence of LBW, improve the awareness rate of knowledge related to gestational hypertension, increase the awareness of pregnancy health care for pregnant women with GDM (22), promote self-monitoring of blood pressure, and promote maternal and child health. Urinary protein should also be routinely monitored in pregnant women with GDM in the prenatal clinic.

### Weight gain during pregnancy

As the obesity epidemic continues, many physicians are interested in minimizing gestational weight gain for all women. High rates of gestational weight gain, especially in the first trimester, are associated with an increased risk of gestational diabetes mellitus (23). Studies on the relationship between GDM and neonatal weight have focused more on macrosomia (24). This study revealed that inadequate GWG was a risk factor for LBW infants, but no association was found between excessive GWG and LBW infants. A recent retrospective study in China showed that women with inadequate weight gain were 2.48 times (10) more likely to deliver low LBW than those with adequate weight gain, and the same retrospective study in Portugal was 1.36 times more likely (25). Our study has similar results. In this study, the proportion of pregnant women who experienced insufficient weight gain during pregnancy was 55.70%, which was similar to the results of a Korean study (26). Pregnant women diagnosed with GDM pay more attention to weight control, including diet and physical activity, during the remainder of their pregnancy (27), but they should be aware that an overcontrolled diet can lead to LBW.

Therefore, it is necessary to formulate gestational weight control guidelines for pregnant women with GDM based on prepregnancy BMI, carry out a “gestational diabetes specialist clinic,” guide the diet and exercise intervention of pregnant women with GDM, strengthen blood glucose monitoring, and control pregnancy weight within a reasonable range.

### Triglycerides in early pregnancy

Maternal triglycerides (TGs) are important for intrauterine development (28). TGs mainly play a role in saving energy providing energy to the human body and participating in energy metabolism (29). Lipogenesis and fat accumulation are enhanced in early pregnancy to prepare for rapid neonatal growth during later gestational stages. Fatty acids derived from maternal plasma TG can promote the placental expression of insulin-like growth factors to promote intrauterine fetal growth (30). Free fatty acid levels increase during the first and second trimesters of normal pregnancy and return to normal during the third trimester. High TG levels can promote the transport and decomposition of lipids by the placenta, accelerate the synthesis of amino acids and proteins, promote the deposition of fat in the fetus, and lead to macrosomia. It was found that maternal lipid levels in early gestation correlated with neonatal birth weight (31). Previous studies have also shown that high maternal TG levels are associated with increased birth weight in European and Chinese populations (32). Another study in China showed that the birth weight of newborns in the GDM group was correlated with TG, and the correlation coefficient (*r* = 0.604; *p* < 0.05) was not significant in the non-GDM group. It is speculated that TG can be used to monitor advanced GDM (33). This study revealed that elevated TG levels in the first trimester of pregnancy in women with GDM were a protective factor against LBW in neonates. It is suggested that pregnant women diagnosed with GDM need to develop a refined diet control plan according to the TG level in the first trimester to control triglycerides at a reasonable level.

### Threatened preterm birth

This study revealed that threatened preterm birth was a risk factor for LBW in pregnant women with GDM, which is consistent with the results of previous studies (34). Therefore, when pregnant women have irregular contractions, vaginal water, and other symptoms of threatened preterm birth, we should be alert to the possibility of preterm birth and delivery of LBW infants and provide timely corresponding medical interventions to improve the fetal protection rate to improve pregnancy outcomes.

### History of fetal distress

This study also revealed that GDM with a history of fetal distress in the previous pregnancy was a protective factor for LBW, which may be due to the experience of a previous adverse pregnancy, leading to increased awareness of pregnancy health care in the next pregnancy, thereby reducing the incidence of LBW. This topic remains to be further analyzed in the future.

### Nomogram

The nomogram is an easy, convenient, and quick tool that can be used to predict the likelihood of delivering LBW after a pregnant woman has been diagnosed with GDM. Nomograms can individually estimate the probability of LBW by integrating various risk predictors, which meet our desire for visual tools and fulfill our drive toward personalized prevention. Through a user-friendly digital interface, improved accuracy, and easier-to-understand risk predictions compared to traditional mathematical formulas, rapid computational nomograms can seamlessly integrate risk assessment into clinical decision-making (35). In recent years, nomograms have been gradually applied to obstetrics and gynecology and maternal and child health care in China, for example, Chuangchuang Xu et al. (36) developed a prediction model for early postpartum stress urinary incontinence in women who had vaginal deliveries, and Mei Kan et al. (37) developed a model for identifying GDM in early pregnancy.

With the help of these simple, rapid, inexpensive, and noninvasive tools, we expect to be able to effectively identify individuals with a potentially increased risk of LBW delivery among pregnant women with GDM. For pregnant women with GDM who are at risk of delivering infants with LBW, medical staff need to be more cautious in medical intervention, pay attention to the intrauterine growth and development of their newborns, and provide targeted prenatal care.

### Research implications

In conclusion, women with GDM are likely to birth LBW infants. This study confirms the necessity and feasibility of assessing LBW at delivery in all pregnant women with GDM. Using the nomogram, physicians in the obstetrics clinic can quickly and directly identify pregnant women with GDM who are at risk for LBW. Based on this, personalized health interventions are initiated, which can help improve neonatal outcomes.

### Strengths and limitations of the study

Overall, our findings provide potential predictive factors for the LBW of infants born to GDM mothers, and the developed risk prediction model facilitates the visual representation of these risks, which is beneficial for clinical application and dissemination. This model also offers useful guidance and practical value for the early identification of high-risk GDM mothers with LBW infants.

The findings of this study should be considered in the context of the following limitations. First, we collected data from only one hospital, and the results are not nationally representative and may not apply to all women in China or elsewhere. Second, the nomogram must be tested for its validation due to its first development. Third, this study was based on a retrospective case–control study to construct a risk prediction model. Some potential confounding factors were not collected, such as smoking, baseline diet, exercise, and psychological indicators in pregnant women.

## Conclusion

This study developed a prediction model and risk score for LBW infants who were born to pregnant women with GDM in Southeast China.

## Data Availability

The raw data supporting the conclusions of this article will be made available by the authors, without undue reservation.
